# Relationship of Soft Tissue Thickness With Body Mass Index and Perioperative Factors in Patients Undergoing Total Hip Arthroplasty: A Retrospective Cross-Sectional Study

**DOI:** 10.7759/cureus.77581

**Published:** 2025-01-17

**Authors:** German R Acosta Gomez, Roberto Acosta Gomez, Maria Del Carmen Garcia Ruiz, Antonio García Hernandez, Atanacio Lopez Valero, Felipe M Camarillo Juarez

**Affiliations:** 1 Orthopaedics, Hospital General de Mexico "Dr Eduardo Liceaga", Mexico City, MEX

**Keywords:** bmi, hip, joint replacement, risk factors, total hip arthroplasty, total joint arthroplasty

## Abstract

Background

Total hip arthroplasty is one of the most common elective procedures, making it crucial to minimize associated risks. The thickness of soft tissues at the surgical site is used to predict complications when evaluated individually. However, the direct relationship between soft tissue thickness, body mass index (BMI), and perioperative factors has not been established, making it necessary to determine this relationship to improve the prediction of complications using only standard radiographic measurements. The objective of this study is to determine whether there is a significant correlation between soft tissue thickness, BMI, and perioperative factors in patients who have undergone total hip arthroplasty.

Methods

A total of 106 records from the Orthopedics and Traumatology service at the General Hospital of Mexico “Dr. Eduardo Liceaga” were analyzed, of which 88 patients met the inclusion criteria. A retrospective, cross-sectional, observational, and descriptive study was conducted using these complete records of patients who underwent primary total hip arthroplasty from 2020 to 2023. Radiographic measurements were performed to determine soft tissue thickness using the Bernaus technique. These measurements were then compared with collected data, including weight, height, BMI, glucose levels, blood loss, and surgical time.

Results

In our statistical analysis, we find a moderate correlation of 0.552 between BMI and soft tissue thickness, indicating a positive association. The analysis showed that an increase in BMI is significantly associated with an increase in soft tissue thickness (p = 0.002). No significant relationship was found between soft tissue thickness and sex (p = 0.546) or age (p = 0.666). However, a significant relationship was observed between surgical time and patient age (p = 0.023), suggesting that the duration of the procedure increases with age. No significant relationships were found between surgical time and BMI, glucose, or weight.

Conclusion

The study confirms a significant positive association between soft tissue thickness and BMI, suggesting that greater soft tissue thickness correlates with a higher BMI. These findings highlight the importance of BMI in evaluating soft tissue thickness, which could influence surgical planning. However, soft tissue thickness shows limited association with certain perioperative factors in total hip arthroplasty patients. These findings emphasize the need for further research with more detailed variables and larger samples to validate these results and enhance recommendations for total hip arthroplasty procedures.

## Introduction

Worldwide, it is estimated that 240 million people suffer from symptomatic osteoarthritis that limits their activity [1]. Total hip arthroplasty (THA) is one of the top ten elective procedures performed in orthopedics [2], and 43% of the 54 million people in the US live with daily life limitations due to this condition [3].

Body mass index (BMI) is a measure of the association between weight and height, calculated using the formula “BMI = kg/m²,” and is used as a predictor for postoperative complications in THA [4]. Patients with obesity are more likely to undergo joint surgery due to a higher prevalence of osteoarthritis compared to those with a normal or overweight BMI [5].

Bernaus et al. describe measuring hip soft tissue thickness by assessing the distance from the greater trochanter tip to the skin, following a line perpendicular to the femoral diaphysis on an anteroposterior radiograph in a supine position with internal rotation of 15-25° [6]. Sprowls et al. measure soft tissue thickness from the highest point of the greater trochanter to the skin edge on standing hip radiographs taken one year after surgery [7].

Bell performs three measurements: from the superior acetabular rim to the skin surface, from the tip of the greater trochanter to the skin surface, and from the most prominent lateral side of the greater trochanter to the skin surface. However, Bell's study did not show any association between peritrochanteric fat thickness and complications such as infections or wound issues after THA [8].

Complications during THA can arise if risk factors are not properly identified, making it crucial to find tools or predictors to minimize these risks. With rising obesity rates, investigating the relationship between BMI, soft tissue thickness, and perioperative factors is essential.

While soft tissue thickness has been linked to complications in other joints, its relationship with BMI and perioperative factors like bleeding and surgical time remains unexplored in THA patients. Understanding this connection could provide a practical tool for predicting risks using standard radiographic measurements available in most hospitals.

The study aimed to evaluate the relationship between soft tissue thickness and BMI, as well as its correlation with perioperative factors in THA patients. Radiographic measurements were performed using the Bernaus technique to analyze these variables. The hypothesis proposed that soft tissue thickness at the surgical site is significantly associated (p < 0.05) with BMI and perioperative factors, including surgical time, intraoperative bleeding, and preoperative glucose levels, using a multiple linear regression model.

## Materials and methods

The study conducted is a retrospective, cross-sectional, observational, and descriptive study. The studied population was collected from patient records from the Orthopedics Department at the General Hospital of Mexico “Dr. Eduardo Liceaga” in Mexico City, which included diagnoses of grade IV coxarthrosis and anteroposterior pelvis radiographs of patients who underwent primary total hip arthroplasty.

For our study, we included patient records with a diagnosis of grade IV coxarthrosis or hip fractures who underwent primary total hip arthroplasty at our institution between 2020 and 2023. We required complete clinical records containing radiographic measurements, and perioperative data such as surgical time, intraoperative bleeding, and preoperative glucose levels. Eligible patients were aged 18 years or older with a BMI between 15 kg/m² and 40 kg/m². Surgical times had to be between 30 and 240 minutes, intraoperative bleeding between 50 ml and 1500 ml, and preoperative glucose levels between 50 mg/dL and 300 mg/dL. Records also needed to include an anteroposterior hip radiograph taken in the supine position with 15-25° internal rotation using the Carestream platform.

Our exclusion criteria encompassed records without a diagnosis of grade IV coxarthrosis or hip fractures, those not operated on between 2020 and 2023, and incomplete records lacking necessary information such as radiographic measurements, surgical time, intraoperative bleeding, and preoperative glucose levels. Additionally, records were excluded if the sex of the patient was undefined or if the patient was under 18 years of age. We also excluded records with a BMI less than 18.5 kg/m² or greater than 40 kg/m², surgical times outside the range of 30 to 240 minutes, intraoperative bleeding less than 50 ml or more than 1500 ml, and preoperative glucose levels below 50 mg/dL or above 300 mg/dL. Lastly, records were excluded if they lacked properly positioned radiographs or if anatomical conditions prevented accurate radiographic measurement.

For this study, a sample size of records from patients who underwent THA from 2020 to 2023 and had a diagnosis of hip coxarthrosis was analyzed, resulting in 106 patients. Clinical records were reviewed, and data on BMI, sex, weight, height, age, glucose, bleeding, and surgical time were collected. Radiographic measurements of anteroposterior pelvis views were taken from the available records measuring hip soft tissue thickness by assessing the distance from the greater trochanter tip to the skin, following a line perpendicular to the femoral diaphysis on an anteroposterior radiograph in a supine position with internal rotation of 15-25°.

For our statistical analysis, we used the STATA/MP 14.0 software (StataCorp LLC, College Station, TX, USA). We assessed the relationship between soft tissue thickness and surgical time as dependent variables and examined their associations with BMI, glucose levels, bleeding, and surgical time as independent variables. Additionally, we included demographic factors such as sex, weight, height, and age in our analysis.

## Results

For this study, 106 records from the Orthopedics and Traumatology Department of the Hospital General de México "Dr. Eduardo Liceaga" were analyzed, of which only 88 patients fulfilled the inclusion criteria. Data were analyzed using STATA/MP 14.0. This study focused on exploring the relationship between soft tissue thickness, BMI, and perioperative factors in patients undergoing total hip arthroplasty.

In the demographic data of the sample, 88 patients were studied, including 30 males (34% of the total sample) and 58 females (65.9%). The average age of the patients was 61.2 years, with a minimum age of 27 and a maximum of 88. The average weight was 69 kg, and the average BMI was 26.71 kg/m². The average measurement of soft tissue thickness was 61.75 mm. The average preoperative glucose level was approximately 108 mg/dL, and the average intraoperative bleeding was 421 cc. The surgical approach used was direct lateral in 52 patients (59.09%) and posterolateral in 36 patients (40.9%). Table 1 shows a summary of the variables obtained from the clinical records.

**Table 1:** Summary of variables obtained in patients undergoing total hip arthroplasty Obs: patients; BMI: body mass index

Variable	Obs	Mean	Std Err	95% CI
Age (years)	88	61.7	11.9	27 - 88
Weight (kg)	88	68.6	13.2	43 - 100
Height (cm)	88	159.8	8.4	142 - 181
BMI (kg/m^2^)	88	26.7	3.9	17.9 - 37.1
Soft tissue thickness (mm)	88	61.7	14.5	30 - 97
Glucose (mg/dl)	88	108.2	36.8	71 - 269
Bleeding (ml)	88	421.0	248.6	100 - 1400
Surgical time (min)	88	110	25.7	60 - 200

A multivariable regression was performed to assess the relationship between surgical time and soft tissue thickness concerning various variables such as sex, age, BMI, glucose, and weight.

Regarding soft tissue thickness, a significant relationship was observed between soft tissue thickness and BMI, with a p-value of 0.002, suggesting that an increase in BMI is associated with an increase in soft tissue thickness (Table 2).

**Table 2:** Summary of multivariable regression of soft tissue thickness in patients undergoing total hip arthroplasty *= p<0.05; BMI: body mass index

Soft Tissue Thickness	F	df	R^2^	p-value	95% CI	
Sex	2.3	-3.8	-0.61	0.54	-10.09	5.37
Age	-.04	0.1	-0.43	0.66	-0.27	0.17
BMI	2.3	0.7	3.16	*0.002	0.88	3.89
Glucose	0.03	0.07	0.43	0.67	-0.12	- 0.19
Weight	-0.65	0.5	-1.22	0.22	-1.73	0.41

No significant relationship was found between soft tissue thickness and sex (p = 0.546) or age (p = 0.666), indicating that these variables do not have a significant impact on soft tissue thickness in our studied sample.

Regarding surgical time, the analysis showed a significant relationship between surgical time and patient age, with a p-value of 0.023, suggesting that older patients tend to have longer surgical times (Table 3).

**Table 3:** Summary of multivariable regression of surgical time in patients undergoing total hip arthroplasty * = p<0.05; BMI: body mass index

Variable	Coefficient	Std. Error	t-value	p-value	95% CI Lower	95% CI Upper
Sex	-1.45	7.99	-0.18	0.85	-17.36	14.45
Age	-0.53	0.23	-2.32	*0.02	-0.99	-0.07
BMI	0.92	1.55	0.6	0.55	-2.16	4.02
Glucose	0.03	0.07	0.43	0.67	-0.12	0.19
Weight	-0.65	0.54	-1.22	0.22	-1.73	0.41

No significant relationships were observed between surgical time and the other independent variables, such as sex, BMI, glucose, and weight. The Pearson correlation between BMI and soft tissue thickness was 0.552, indicating a moderate and positive correlation. This finding reinforces the idea that BMI is an important factor in determining soft tissue thickness, which could have clinical implications for surgical planning.

## Discussion

The rise in total hip arthroplasty cases in the population necessitates the search for better predictors to support the development of safe surgeries and anticipate potential complications that could be avoided with an appropriate preoperative protocol. The increase in obesity in our country compels us to make early decisions and evaluate which variables are affected by increased soft tissue thickness.

Obesity has been shown to increase the likelihood of hospital readmission in postoperative THA patients due to complications such as wound infection, sepsis, or thromboembolism, both cardiac and respiratory [9,10]. It also leads to greater dependence on daily activities [11] and increased anesthesia time, operative time, and length of hospital stay [12,13]. Soft tissue thickness at the surgical site has also been used as a predictor of complications in THA. While elevation is known to predispose to postoperative complications, the independent study of soft tissue thickness in relation to perioperative factors remains unclear.

Soft tissue measurements are easily obtained from preoperative hip X-rays and allow for the measurement of soft tissue thickness, which can provide valuable information and assess changes in perioperative factors. Our study observed that increased soft tissue thickness is associated with a higher BMI. This is noteworthy as Rey Fernández et al. reported that elevated soft tissue thickness measured radiographically is associated with an increased risk of periprosthetic infection in patients undergoing total hip arthroplasty [14]. Our study suggests that patients with a high BMI may also be predisposed to a higher risk of periprosthetic infection.

Additionally, Parikh et al. supported the hypothesis that increased soft tissue thickness can predict surgical complications after hip arthroplasty, potentially better than BMI [15]. Wu et al. found that, in reverse shoulder arthroplasty, soft tissue thickness was related to surgical time, hospital stay, and infection [16]. Based on our results, hip surgeons might consider strategies to reduce intraoperative alterations for patients undergoing total hip arthroplasty.

Our research confirms a significant and positive association between soft tissue thickness and BMI. This supports the idea that a higher BMI is linked to increased soft tissue thickness. This analysis and result are important for the preoperative management of patients, as measuring soft tissue thickness with an X-ray can provide insights into how perioperative factors may be altered.

Wagner et al. reported a significant association between BMI and outcomes after knee arthroplasty, including reoperation, prosthetic revision, and complications [17]. In primary knee arthroplasty, reoperation, revision, or implant removal rates are strongly associated with BMI [18,19], and increased anterior subcutaneous fat thickness is associated with a significantly higher risk of reintervention due to wound complications [20]. In lumbar surgery, subcutaneous fat thickness is suggested to be a more significant and accurate marker for surgical site infection [21,22]. Regarding the ankle, radiographic soft tissue thickness is mentioned as a potential risk factor for the need for ankle prosthesis revision [23].

In hip surgeries, no relationship has been observed between soft tissue thickness and perioperative bleeding, a major perioperative factor that may later require blood transfusion [24]. Although blood transfusions account for only a small percentage of overall complications, they are a costly complication for health systems [25]. There is limited evidence in many literature reviews about complication rates among different surgical approaches [26,27]. Diabetic patients or those with uncontrolled blood glucose who undergo joint replacement procedures have a higher risk of early medical complications, but no specific relationship with soft tissue thickness has been established [28].

Our findings suggest a negative relationship between age and surgery duration, indicating that older patients may undergo shorter procedures, possibly due to adaptations in surgical practices for this age group. However, this highlights the need for further research to deepen the understanding of the factors involved. Therefore, future studies are needed that consider a greater variety of variables to gain a more complete understanding of the elements affecting both bleeding, surgery duration, and soft tissue thickness.

Additionally, the results may reflect the impact of comorbidities and anatomical changes in older patients, which necessitate personalized surgical approaches and influence procedure duration. Soft tissue thickness measurements can be valuable in preoperative planning by anticipating equipment needs, extended operative times for higher BMI patients, and potential challenges with surgical exposure and implant placement, ultimately improving safety and outcomes.

There are several limitations to our study. These include the relatively small sample size and the fact that it was conducted at a single institution, which limits the generalizability of the results. Another limitation is the lack of standardization in variables such as age and perioperative factors, which may introduce variability in the results. Future research should involve larger, multicenter studies with a more diverse patient population, and include extended follow-up periods to validate our findings and improve their clinical applicability.

## Conclusions

Our research confirms a significant and positive association between soft tissue thickness and BMI. This supports the notion that increased soft tissue thickness corresponds to a higher BMI. Additionally, soft tissue thickness has a limited association with certain perioperative factors in patients undergoing total hip arthroplasty. However, the findings indicate the need for further research with more detailed variables and larger sample sizes to confirm these results and provide better recommendations for the surgical management of total hip arthroplasties.
